# Assessing the Efficacy and Mechanisms of Pycnogenol^®^ on Cognitive Aging From *In Vitro* Animal and Human Studies

**DOI:** 10.3389/fphar.2019.00694

**Published:** 2019-07-03

**Authors:** Tamara Simpson, Christina Kure, Con Stough

**Affiliations:** Swinburne Centre for Human Psychopharmacology, Swinburne University, Melbourne, VIC, Australia

**Keywords:** Pycnogenol®, aging, cognition, memory, RCT—randomized controlled trial

## Abstract

Brain aging is a complex and multifactorial process broadly involving changes in the brain’s structure, neuronal activity, and biochemical profile. These changes in brain function have also been linked to age-associated variations in cognitive function. Recent research has suggested a role of increased oxidative stress and reduced cognition in older people. Therefore, studies that examine the effects of antioxidants on cognitive performance are important, particularly in the context of an increase in elderly populations in most Western countries. One such antioxidant, Pycnogenol, is a standardized plant-based extract obtained from the bark of the French maritime pine and has a long historical use to treat inflammation and improve health. More recently, Pycnogenol has been subjected to more than 100 research trials. *In vitro* and animal studies using the standardized extract have indicated a multimodal action of Pycnogenol, and several human studies have shown improvements in cognitive function after chronic administration. In this paper, we review these studies in the context of understanding both biological and cognitive changes due to Pycnogenol and evaluate possibilities of Pycnogenol to improve neurocognitive function.

## Introduction

The purpose of this paper is to present mechanistic and efficacy studies relating to the antioxidative properties of the patented French maritime pine bark extract, trading under the name Pycnogenol^®^ (PYC; *Pinus pinaster* ssp. *atlantica*, Pinaceae: Horphag Research Ltd., UK, Geneva, Switzerland) and how these mechanisms support cognitive function in aging. PYC is utilized as a nutritional supplement and as a bioactive remedy for several chronic diseases. Human clinical trials have demonstrated that PYC enhances domains of cognitive ability, and *in vitro* and *in vivo* animal and human studies have assessed its mechanisms of action, particularly its strong antioxidative properties as well as anti-inflammatory and vascular functions. Cost-effective interventions with therapeutic potential to sustain and maintain an individual’s cognitive ability and to alter the biochemical profile and symptom severity in different disease states have stimulated interest within the scientific community and are important targets, particularly for older populations.

### What is Pycnogenol?

**Figure 1:**
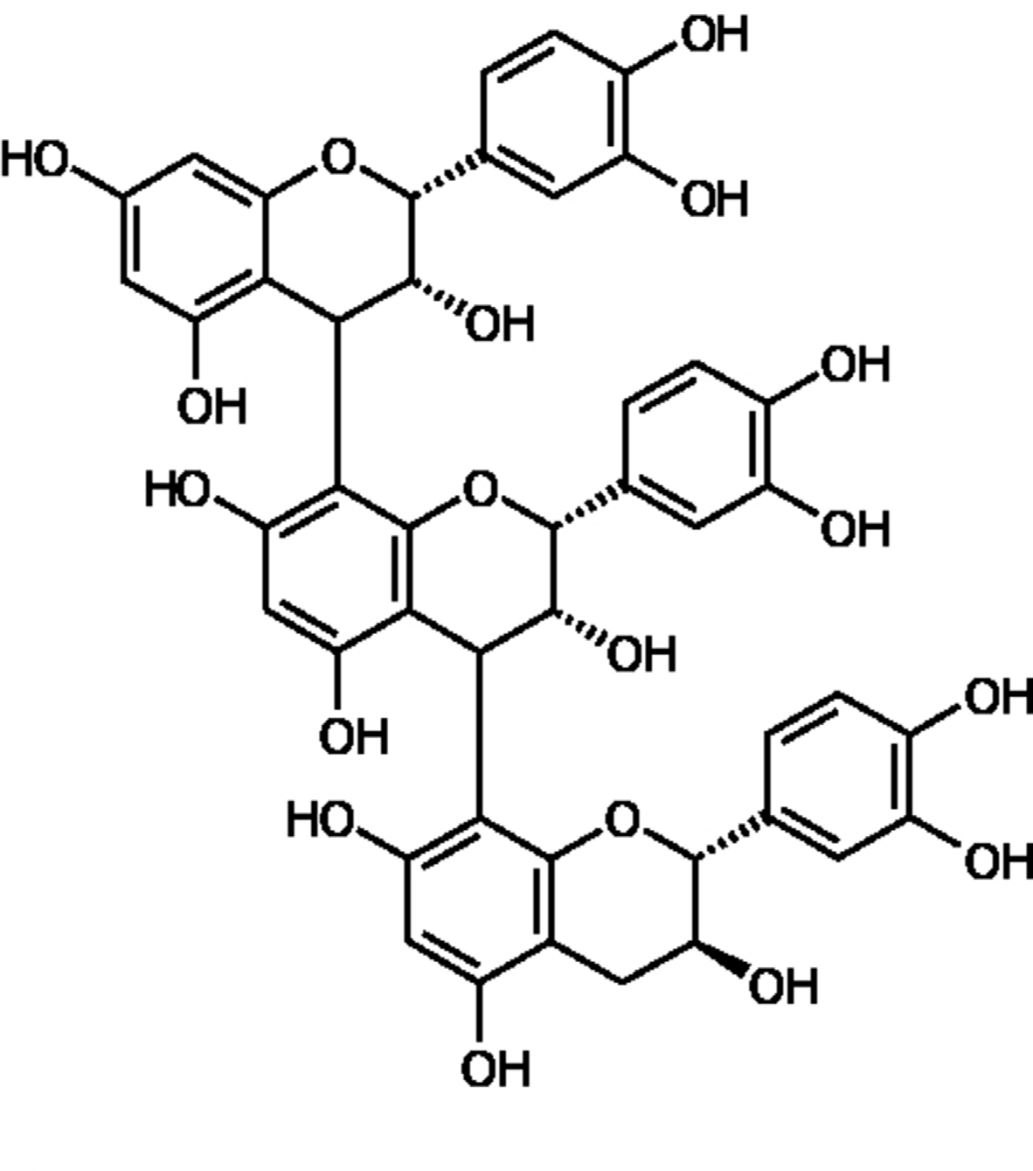
Structural representation of a procyanidin molecule consisting of catechin and epicatechin subunits which are the main constituents of Pycnogenol (PYC). The catechin and epicatechin units can be linked by different bonds, the main being C4-C8 bonds and C4-C6 bonds as well as C4-C8 bonds with different chain lengths up to dodecamers.

PYC is a plant-based extract derived from the bark of the French maritime pine. It grows along the coastal southwest of France. Pine bark extracts have been used traditionally and date back at least to Hippocrates (inflammation) around 400 bc and Hans Minner around 1479 to treat wounds (Drehsen, 1999). The standardized extract is composed of phenolic compounds, divided between monomers (catechin, epicatechin, and taxifolin), condensed flavonoids (classed as procyanidins/proanthocyanidins) and phenolic acids (cinnamic acids and other glycosides) (Petrassi et al., 2000; Rohdewald, 2005; refer to **Figure 1**). After being ingested, these phenolic compounds undergo biotransformation and are broken down in the colon by microbial enzymes yielding smaller, bioavailable molecules that can then be absorbed by the colon into the bloodstream and transported to tissues and organs (Trebatická and Ďuračková, 2015). The nutritional preparation is extracted from crushed bark, followed by a patented extraction process (Rohdewald, 2002). The quality and purity of the raw bark are assessed by a French independent regulatory and control body, The French Association of Norms Association Française des Normes, AFNOR (Association Française des Normes, AFNOR: Packer et al., 1999) and conforms to the monograph “Maritime pine extract” in the United States Pharmacopeia (USP). PYC is available in most countries as an over-the-counter product, in tablet or capsule form in doses varying from 20 to 100 mg (Rohdewald, 2002), and has demonstrated good tolerability with very few side effects and a high level of safety (Rohdewald, 2005). Based on a recent update by the American Botanical Council of the Scientific and Clinical Monograph for Pycnogenol (The American Botanical Council, 2019), it is stated that an independent panel of toxicology experts has classified PYC as generally recognized as safe (GRAS) based on clinical safety and preclinical toxicology data. Additionally, while there are no known contraindications for PYC, it is recommended that children under 6 years of age should not use PYC, and PYC should not be taken during the first 3 months of pregnancy as a precaution. The data of 91 human clinical studies (6,849 people) have informed the safety PYC whereby the frequency of adverse events (AEs) is 2.4%; and in healthy participants, the global AE rate is 0.1%. Furthermore, the AEs reported in the clinical trials were unrelated to the duration of use or dose of PYC. The most commonly reported AE was gastrointestinal discomfort; it is therefore recommended that PYC is taken with food. Other reported AEs are dizziness, headache, and nausea.

Because of its composition of multiple constituents, PYC has several actions that give rise to its reported multiple health benefits. PYC has been widely studied and has shown to have promising effects in improving conditions including diabetes (Spadea and Balestrazzi, 2001; Liu et al., 2004a; Liu et al., 2004b), cardiovascular health (Fitzpatrick et al., 1998; Liu et al., 2000; Valls et al., 2016), asthma (Hosseini et al., 2001; Lau et al., 2004; Shin et al., 2013), osteoarthritis (Farid et al., 2007; Belcaro et al., 2008a; Belcaro et al., 2008b), sexual disorders (Stanislavov and Nikolova, 2003; Aoki et al., 2012; Kobori, 2015), venous insufficiency (Petrassi et al., 2000; Cesarone et al., 2010; Gulati, 2014), and neurological disorders including attention-deficit hyperactivity disorder (Chovanová et al., 2006; Dvořáková et al., 2006; Trebatická et al., 2006) and cognitive impairment (Maimoona et al., 2011). For the purposes of this review, we characterize the antioxidative activity of PYC, how this activity relates to aging, and how these mechanisms support cognitive function.

### Cognitive Function and Oxidative Stress in the Aging Brain

Cognition is a collective term generally referring to mental processes requiring the ability to remember and recall information and use information logically as well as abstractly and tasks that require sustained attention (Budson and Price, 2005). While cognitive impairment or deterioration is a hallmark of advancing age, the outcomes are more insidious where there are functional losses that impact an individual’s potential to lead an active, productive, and healthy lifestyle. Alterations in brain structure, neural activity, and biochemical profile are believed to contribute to the associated functional losses of reduced cognitive ability (Sohal and Orr, 2012). Particularly, an increase in oxidative stress, elevated inflammation, and damage to the vasculature all play a role in age-associated cognitive decline (Teunissen et al., 2003; Ansari and Scheff, 2010; Jomova et al., 2010; Waldstein and Elias, 2015). Oxidative stress is an imbalance in the production of free radicals [reactive oxygen species (ROS)] and the antioxidant system. In the elderly, age-related oxidative stress is multifactorial, marked by increased production of free radicals, reduced activities of antioxidant enzymes, diminished antioxidant levels, and weakened repair of oxidative damage (Rybka et al., 2011). During aging, the presence of persistent oxidative stress and lack of antioxidant support increases the production of amyloid-β plaques in the brain, increasing the risk of developing neurodegenerative disease, mild cognitive impairment, and Alzheimer’s disease (AD) (Smith et al., 2002; Mariani et al., 2005).

Aging impacts the brain’s capacity to scavenge free radicals such as ROS because of reduced antioxidant defenses, making oxidative stress a significant feature of general aging and age-related neurodegenerative disorders like AD (Cadenas and Davies, 2000; Valko et al., 2007). Increased production of ROS with or without decreased antioxidant defenses leads to damage of macromolecules, impaired organ function, and disease development (Finkel and Holbrook, 2000). The human brain is highly susceptible to oxidative damage. This is due to several factors, particularly because of its 1) high oxygen metabolic activity. The human brain consumes 20% of the body’s oxygen metabolism, providing energy for neuronal activity (Raichle et al., 2001). This dynamic metabolic exchange must be stable for normal brain function. Dysfunctional oxygen metabolism is the hallmark of neurodegenerative disorders associated with cognitive decline (Watts et al., 2018); 2) high concentration of polyunsaturated fatty acids (PUFAs). The biochemical process of excessive free radical-mediated peroxidation of PUFA causes destruction to cell membranes, structure, and function (Syslová et al., 2014); 3) cytotoxic action of the excitatory amino acid glutamate. Glutamate receptors are found on the surface of cells and therefore can only exert its excitatory function through extracellular fluid (Zhou and Danbolt, 2014). If glutamate receptors are excessively activated, the increased glutamate can overstimulate nerve cells, depolarizing them (known as excitotoxicity) and eventually leading to cell death (Kritis et al., 2015); 4) compared with other organs of the human body, the brain has reduced antioxidant mechanisms due to lower glutathione peroxidase (GPx) and catalase (CAT) (Packer, 1992). Both GPx and CAT reduce hydrogen peroxide into water and oxygen to assist in maintaining beneficial levels of ROS to prevent damage to molecular bonds, cell membranes, and DNA (Basu, 2010).

Additionally, essential biometals such as copper, iron, and zinc are involved in several biological processes and are essential for the survival of living organisms. These biometals, particularly iron and copper, act by increasing the speed of chemical reactions due to their oxidizing and reducing powers (redox potential) (Greenough et al., 2013). It is because of this process, where oxygen is transported and/or metabolized, that free radical formation (superoxide and hydroxyl radicals) and other ROS, formed *via* the Fenton reaction, increases the risk of oxidative stress in the brain (Winterbourn, 1995; Jomova and Valko, 2011). This breakdown or dyshomeostasis of biometals whereby antioxidant protection is overwhelmed in favor of elevated concentration of metals leads to a state of neurotoxicity with damaging interaction to proteins, lipid peroxidation, and DNA, all evidence of increased oxidative stress in the AD affected brain (Jomova et al., 2010; Jomova and Valko, 2011). Oxidative stress has been suggested to be not only an early feature of AD but also the mechanism that leads to the disease state (Ansari and Scheff, 2010). The cognitive impairments experienced by those with AD severely impact the individual’s ability to function on a daily basis. They are unable to remember new information and perform complex tasks, have problems with language functions as well as visuospatial abilities, and lose the ability to make good judgments (McKhann et al., 2011).

Due to these mechanisms of aging and hallmarks of neurodegenerative diseases, it may be beneficial for older individuals to consume dietary interventions with antioxidant properties to maintain cognitive function and reduce the risk of increased oxidative stress (Simpson et al., 2015). In light of this, the antioxidant mechanisms of PYC are discussed below.

### Antioxidative Activity of Pycnogenol

Due to its antioxidant and various biomodulating effects, PYC has potential benefits in improving cognitive function because it acts as a regulator and protects cells against oxidative stress by 1) being a potent free radical scavenger; 2) protecting DNA from damage; 3) increasing the synthesis of antioxidant enzymes; and 4) protecting other endogenous antioxidants (vitamin C, vitamin E, and glutathione) from oxidative damage (Packer et al., 1999; Rohdewald, 2002). Demonstrating its antioxidant actions, PYC had been shown to decrease oxidative damage to DNA in children with attention deficit hyperactivity disorder (Chovanová et al., 2006). In a group of patients suffering from erectile dysfunction, plasma antioxidant activity significantly increased total cholesterol, and low-density lipoprotein cholesterol decreased in response to PYC intervention (Ďuračková et al., 2003). Experiments on cultured neuroblastoma cells exposed to oxidative stress toxicity (acrolein induced) have showed that pre-treatment with PYC significantly diminished the ensuing cytotoxicity, protein damage, lipid peroxidation, and cell death (Ansari et al., 2008). Furthermore, in animal models of dementia, PYC and vitamin E mitigated cognitive deficits and oxidative damage in the cerebral cortex and hippocampus (Ishrat et al., 2009). Clinical studies have demonstrated the antioxidant function of PYC. For example, a 6-week PYC intervention (150 mg/day) in healthy adults resulted in a significant increase in plasma oxygen radical absorbance capacity (ORAC), a biomarker of antioxidant activity (Devaraj et al., 2002). Additionally, in smokers who largely have higher oxidative stress than non-smokers, PYC supplementation (50 mg/day) reduced plasma levels of reactive oxidative species (derivatives of the reactive oxygen metabolites test) and increased antioxidant potential (biological antioxidant potential test) (Belcaro et al., 2013).

Researchers have also revealed additional pharmacological actions that may benefit conditions associated with cognitive impairments. One study showed that PYC not only exerts antioxidant activity but also increases plasma high-density lipoprotein and decreases serum low-density lipoprotein, indicating additional benefits on the lipid profile (Stough et al., 2012b). A study of erythrocyte membrane fluidity found increased membrane surface fluidity in response to PYC as well as a further outcome of protection against lipid peroxidation either from chelating metal ions or quenching free radicals, or both (Sivonová et al., 2004). As it is known that lipoproteins are associated with cognitive impairments in the elderly (Van Exel et al., 2002), this mechanism may also be important in improving cognition. PYC has also demonstrated a potential role in protecting genetic damage through its antimutagenic effects (Križková et al., 2008). Attenuation of DNA cleavage has been seen in beta-amyloid-induced apoptosis in rodent models (Peng et al., 2002). However, since research into the genetic effects of PYC is in its infancy, these findings need to be further explored to determine the clinical use of these genetic mechanisms. Findings from these studies further support the multiple roles PYC may have as a potential cognitive enhancer (Liu et al., 2000).

Mechanistically, PYC targets free radicals derived from both oxygen and nitrogen. While at higher concentrations nitric oxide (NO) is detrimental and contributes to neuroinflammation and neurodegeneration, at normal physiological levels, the role of NO is integral within the brain for neurotransmission (modulates serotonin, dopamine, and norepinephrine), neuromodulation, and synaptic plasticity (Dvořáková et al., 2006). The balance of NO production within the brain is therefore integral to cognitive health. Animal experiments have demonstrated that learning and memory increase NO production within the brain (Paul and Ekambaram, 2011). PYC either inhibits or increases vasodilation by assisting in the regulation of endothelial cell production of NO, which increases blood flow as well as oxygenation and transport of nutrients around the body to all organs including the brain (Fitzpatrick et al., 1998; Finkel and Holbrook, 2000). This action of relaxing blood vessels and making them more flexible not just benefits the brain but also reduces demand on the cardiovascular system and entire body due to better circulation.

The effects of PYC on stimulated macrophage cell cultures to mimic the immune response identified that the most active metabolite of PYC, M1 (δ-(3,4-dihydroxyphenyl)-γ-valerolactone), mediated anti-inflammatory effects by impeding nitric oxide generation and inducible nitric oxide synthase (iNOS) upregulation production (Uhlenhut and Högger, 2012). Furthermore, M1 has been identified to accumulate in erythrocytes, monocytes, and endothelial cells with this cellular uptake and has the ability to cross the blood–brain barrier (BBB) possibly *via* the GLUT-1-type glucose transporter (Uhlenhut and Högger, 2012; Kurlbaum et al., 2013). The M1 metabolite is a by-product of PYC consumption formed in the gut from catechins (Kurlbaum et al., 2013). An *in vitro* study utilizing cultured B16 melanoma cells found that while PYC suppressed reactive species in their assays, glutathione was enhanced (Kim et al., 2008). The most important intracellular antioxidant in the human body is glutathione (GSH). GSH is a unique tripeptide made from glutamate, cysteine, and glycine found predominantly in the cytosol, mitochondria, and cell nucleus (Berk et al., 2008). GSH has many roles; for example, it acts as a detoxifier, and the ratio of reduced to oxidized glutathione (GSH:GSSG) forms a marker of intracellular antioxidant redox potential to regulate drug detoxification and elimination; second, GSH’s antioxidant properties protect proteins, lipids, and nucleic acids from free radicals, peroxides, and toxins (Wu and Batist, 2013). Interestingly, the facilitated uptake of M1 into cells involves further metabolism by conjugation with GSH in erythrocytes (Kurlbaum et al., 2013). Therefore, the antioxidative properties of PYC make it particularly novel in reducing oxidative stress in the brain than do other endogenous antioxidants, which are unable to cross the BBB (Iuga et al., 2011).

**Figure 2:**
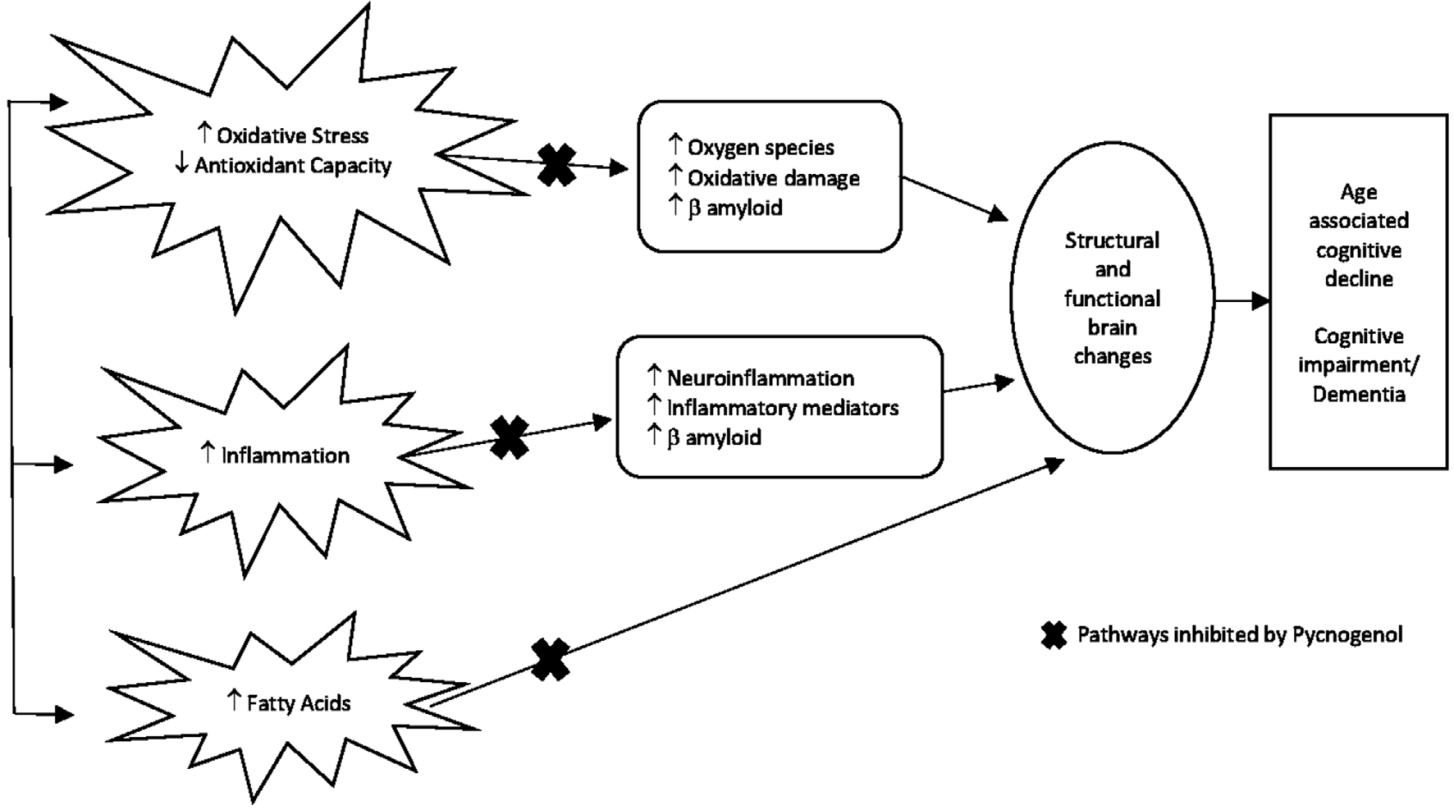
Proposed mechanism of action of PYC as a targeted therapy for preventing cognitive decline. With increasing age, inflammation-reduced antioxidant metabolism leading to increased oxidative stress and damage to fatty acids are common mechanisms that over time can impact on the brain causing structural and functional changes culminating in the outcome of age-associated cognitive decline, cognitive impairment, and/or dementia. PYC potentially inhibits these mechanisms as represented by the x in the diagram due to its scavenging ability to free radicals and protection of proteins (biomolecules) against oxidative damage (Packer et al., 1999; Rohdewald, 2002; Ansari et al., 2008), neuron protection from β amyloid-induced apoptonis (Peng et al., 2002; Gulati, 2014), anti-inflammatory effects (Lau et al., 2004), and reduction of fatty acids (Sivonová et al., 2004).

Several studies discussed confirm PYC’s antioxidant and free radical scavenging effects and potential use in improving cognitive function (see **Figure 2**). Future studies are required to investigate the modulation of GSH metabolism in response to PYC administration and further elucidate the role of M1 and GSH. Interestingly, neuroimaging studies have not yet been performed as a method to investigate GSH metabolism and cognitive effects in relation to PYC administration. Given that oxidative stress is known to affect neuroplasticity, attempts to normalize such impairments are of great therapeutic value in aging and cognition research. Neuroimaging is a non-invasive modality that may assist in answering structural, functional, and mechanistic questions, complementary to the *in vivo* animal, histological, and hematological studies and human clinical trials that have been discussed in response to PYC intervention. We now review studies that have investigated the efficacy of PYC as a cognitive enhancer in humans.

### Pycnogenol as a Cognitive Enhancer

Preliminary findings from clinical trials show promising cognitive-enhancing effects of PYC in healthy adults. Luzzi et al. (2011) found that in a group of young healthy university students, an 8-week PYC intervention (100 mg/day) when taken in conjunction with a standardized health plan resulted in improvements in sustained attention, memory, and executive functioning. Students in this study who were taking PYC also showed improvements on mood parameters (alertness, contentedness, and reduced anxiety) and performed significantly better on actual university exams than did the control group. In a similar study, Belcaro and colleagues (2014) examined the effects of a 12-week intervention comprising PYC (150 mg/day) and a healthy lifestyle plan (controlled diet and lifestyle suggestions) in young healthy professionals (aged 33 and 55 years) who had elevated oxidative stress at baseline. These authors found significant improvements in those who were taking PYC on mood (alertness and attention) and cognitive parameters (episodic memory and spatial working memory) and a 30.4% reduction in the oxidative stress marker, with no changes seen in controls. Furthermore, a 3-month daily PYC intervention (150 mg/day) resulted in improvements in working memory, but not in concentration or psychomotor abilities, in healthy, elderly individuals and concomitant decreases in plasma levels of the oxidative stress marker F2-isoprostanes (Ryan et al., 2008). Findings from these studies are promising, and trials with larger sample sizes are currently being conducted to confirm these potential benefits of PYC in preventing cognitive decline (Stough et al., 2012b).

## Conclusions

Converging evidence suggests that the biomodulating effects of PYC improve several mechanisms that may underpin cognition including vascular, anti-inflammatory, neuroprotective, and antioxidant processes. Research into PYC for improving cognitive function is growing with preliminary clinical studies indicating benefits on several cognitive domains including attention, memory, and executive functioning. Future clinical studies using PYC in larger sample sizes and over longer treatment conditions are needed to further support its use as a treatment for improving cognitive function and to further elucidate the antioxidant mechanisms supporting cognitive health and/or improvement (Stough et al., 2012a). Research investigating the role of M1 in relation to GSH as well as the effect of PYC on brain metabolites (e.g., Simpson et al., 2019) is an area of study that shows promise in elucidating the *in vivo* brain effects and mechanisms of PYC. Furthermore, neuroimaging techniques to investigate brain structure, function, and metabolism in response to PYC is warranted.

## Author Contributions

TS, CK, and CS all contributed to the design, writing, and editing of the manuscript.

## Funding

The authors thank the Australian Research Council Discovery Grant (DP1093825) to CS, with additional funding from Horphag and philanthropic grants from Doug Mitchell and Roderic O’Connor, while working on this manuscript. TS received an Australian Government Research Training Program Scholarship.

## Conflict of Interest Statement

The authors conduct studies using Pycnogenol^®^ that are funded by Horphag. Horphag had no role in the preparation of the manuscript or decision to publish.
